# Reporting quality of randomized controlled trials in patients with HIV on antiretroviral therapy: a systematic review

**DOI:** 10.1186/s13063-017-2360-2

**Published:** 2017-12-28

**Authors:** Kaori Nagai, Akiko M. Saito, Toshiki I. Saito, Noriyo Kaneko

**Affiliations:** 1 Department of Global and Community Health, Graduate School of Nursing, Nagoya City University, 1 Kawasumi, Mizuho-cho, Mizuho-ku, Nagoya, 467-8601 Japan; 2 Department of Clinical Research Management, Clinical Research Center, National Hospital Organization Nagoya Medical Center, 4-1-1 Sannomaru, Naka-ku, Nagoya, Aichi 460-0001 Japan

**Keywords:** HIV/AIDS, ART, Randomized controlled trials, CONSORT, Reporting quality

## Abstract

**Background:**

To allow for correct evaluation of clinical trial results, readers require comprehensive, clear, and highly transparent information on the methodology used and the results obtained. This study aimed to evaluate the quality of reporting in articles on randomized controlled trials (RCTs) of antiretroviral therapy (ART) in the field of HIV/AIDS.

**Methods:**

We searched for original articles on RCTs of ART developed in the field of HIV/AIDS in PubMed database by 5 April 2016. Searched articles were divided into three groups based on the revision year in which the Consolidated Standards of Reporting Trials (CONSORT) guidelines were published: Period 1 (1996–2001); Period 2 (2002–2010); and Period 3 (2011–2016). We evaluated the articles using the reporting rates of the 37 items in the CONSORT 2010 checklist, five items in the protocol deviation, and the three items in the ethics.

**Results:**

Fifty-two articles were extracted and included in this study. Many of the reporting rates calculated using the CONSORT 2010 checklist showed a significantly increasing trend over the successive periods (65% in Period 1, 67% in Period 2, 79% in Period 3; *p* < 0.0001). The items with reporting rates < 50% were “the presence or absence of a protocol change and the reason for such a change,” “randomization and blinding,” and “where the full trial protocol can be accessed.” Reporting rates of deviations were as low as < 30%, while the reporting rates for patient compliance were the highest (>80% in Period 3) among the five items. The reporting rates for obtaining informed consent and approval by the ethics committee or institutional review board were high (>88%), regardless of the time period assessed.

**Conclusion:**

In terms of representative RCT articles in the field of HIV/AIDS, the reporting rate of the items defined by CONSORT was approximately 70%, improving over the successive CONSORT statement revision periods.

**Electronic supplementary material:**

The online version of this article (doi:10.1186/s13063-017-2360-2) contains supplementary material, which is available to authorized users.

## Background

To allow for correct evaluation of clinical trial results, readers require comprehensive, clear, and highly transparent information on the methodology used and the results obtained. The Consolidated Standards of Reporting Trials (CONSORT) statement [1–3] aims to improve the scientific quality of reports of randomized controlled trials (RCT) and was developed in collaboration with clinical trial methodologists, guideline developers, knowledge translation specialists, and journal editors. This statement evaluates the quality of the reporting of clinical trial results, but not of the clinical trial itself. The first version of the CONSORT statement was developed in 1996 and was thereafter revised twice, in 2001 and 2010 [1–3]. The current CONSORT 2010 statement is composed of a checklist of 25 topics and includes 37 items and a flow diagram. It has been recommended that the statement be used in conjunction with the CONSORT 2010 Explanation and Elaboration [4], which was developed simultaneously with the statement [4].

Systematic reviews evaluating the reporting quality of clinical trials in the field of HIV/AIDS have included various articles. Some reports assessed the quality of reporting of adverse events using the CONSORT 2001 extension for adverse events reporting [5, 6]. Another report targeted studies in which HIV interventions were performed by a pharmacist and their validity was assessed using the CONSORT 2010 statement and the Strengthening the Reporting of Observational Studies in Epidemiology (STROBE) Statement criteria [7]. A further report targeted non-inferiority RCTs [8] and yet another report evaluated the performance and reporting quality of RCTs aimed at prevention and treatment of HIV/AIDS in Africa [9]. However, to our knowledge, no study has comprehensively reviewed and evaluated the quality of reports on RCTs of antiretroviral therapy (ART), the standard therapy for HIV/AIDS using the CONSORT 2010 statement.

The CONSORT statement does not include a detailed protocol deviation item checklist. On the other hand, the report by Sweetman and Doig [10] classified protocol deviations into five items, i.e. enrollment, randomization, study intervention, patient compliance, and data collection. These items were defined as indices for the evaluation of clinical trial quality, with the purpose of examining relationships between protocol deviation reporting and the characteristics of clinical trials. Based on the results of their study, they proposed addition of the five protocol deviation items to the CONSORT statement in order to clarify the requirements for protocol deviation reporting. In the field of HIV/AIDS, compliance with medication is an important index, because it is considered important for sustained HIV suppression and reduction of the risk of drug resistance [11]. Several compliance-related studies have been reported, including a study using a device that informs HIV patients with impaired memory when to take their medication [12] and another study on compliance rate and outcomes [13–17].

According to the 2016 fact sheet of the Joint United Nations Programme on HIV/AIDS (UNAIDS), there were 36.7 million HIV-infected individuals alive as of 2015 and 70% of those people resided in sub-Saharan Africa (19 million people in Eastern and Southern Africa and 6.5 million people in Western and Central Africa) [18]. The majority (33 of 48 countries) of sub-Saharan Africa countries are poorly developed countries [19]; this area includes many regions in which the literacy rate is low [20]. The United Nations defines “least developed country” based on the following three criteria: per capita income; human assets; and economic vulnerability [21]. It has been shown that informed consent processes require more effort in developing countries than in advanced countries [22] and it has been recommended that a local translator write the information pamphlet as well as the consent form and that informed consent should be acquired in the local language [23]. However, there has been no detailed review of the reporting on obtaining informed consent in RCTs in the field of HIV/AIDS.

Thus, this study aimed to evaluate the reporting quality of articles on RCTs of ART developed in the field of HIV/AIDS, by adding evaluation items related to protocol deviations and ethical aspects to the CONSORT 2010 checklist items.

## Methods

### Search strategy

Original articles on ART RCTs that had been reported between the start date of the PubMed database and 5 April 2016, were extracted by means of a PubMed search (Appendix 1 lists the search terms used). Additionally, when an extracted article was not the original article, the original article was manually identified from the reference list of the extracted article.

### Article selection

Article selection criteria consisted of the following. Original articles on RCTs of ART developed in the field of HIV/AIDS were included. Brief reports, rapid communications, short communications, conference abstracts, study protocols, and reports of non-RCTs, such as sub-studies and pilot studies, were excluded. One researcher reviewed all abstracts of the extracted articles to exclude ineligible abstracts. Thereafter, this researcher reviewed full texts to exclude ineligible articles. Another researcher independently reviewed the ineligibility of the excluded articles.

### Data evaluation

One researcher evaluated the full texts and two other researchers independently assessed these evaluations. The four researchers resolved differences in evaluations by means of discussion.

### Setting the evaluation period

The first CONSORT statement was published on August 1996, which was later revised in April 2001 and March 2010. Therefore, we divided the articles into three groups based on the year in which they were published: Period 1 (1996–2001); Period 2 (2002–2010); and Period 3 (2011–2016). Evaluation items were compared the different versions of the checklist (Table 1). In the first revision, item numbers were changed, combined, or split. In the second revision, minor changes were made to item numbers, while major changes involved subdivision of existing topics, which increased the specificity and understanding of the evaluation items. Additionally, a new section, “Other information,” was added, so that the number of sections increased from five to six.

**Table 1:** Changes in CONSORT checklist item

CONSORT 2010				CONSORT 1996	CONSORT 2001	CONSORT 2010
Section	Topic		Checklist item	Item no	Item no	Item no
Title and abstract			Identification as a randomized trial in the title	1	1*	1a^†^
			Structured summary of trial design, methods, results, and conclusions (for specific guidance see CONSORT for abstracts)	2	-	1b^†^
Introduction	Background and objectives		Scientific background and explanation of rationale	-	2	2a
			Specific objectives or hypotheses	3	5*	2b
Methods	Trial design		Description of trial design (such as parallel, factorial), including allocation ratio	-	-	3a
			Important changes to methods after trial commencement (such as eligibility, criteria), with reasons	-	-	3b
	Participants		Eligibility criteria for participants	4	3*	4a*
			Settings and locations where the data were collected	-		4b*
	Interventions		The interventions for each group with sufficient details to allow replication, including how and when they were actually administered	5	4*	5*
	Outcomes		Completely defined pre-specified primary and secondary outcome measures, including how and when they were assessed	6	6*	6a*
			Any changes to trial outcomes after the trial commenced, with reasons	-	-	6b
	Sample size		How sample size was determined	6	7*	7a*
			When applicable, explanation of any interim analyses and stopping guidelines	8		7b*
	Randomization	Sequence generation	Method used to generate the random allocation sequence	10	8*	8a^†^
			Type of randomization; details of any restriction (such as blocking and block size)	9		8b^†^
		Allocation concealment mechanism	Mechanism used to implement the random allocation sequence (such as sequentially numbered containers), describing any steps taken to conceal the sequence until interventions were assigned	11	9*	9*
		Implementation	Who generated the random allocation sequence, who enrolled participants, and who assigned participants to interventions	12	10*	10^†^
	Blinding		If done, who was blinded after assignment to interventions (for example, participants, care providers, those assessing outcomes) and how	13	11*	11a*
			If relevant, description of the similarity of interventions			11b*
	Statistical methods		Statistical methods used to compare groups for primary	7	12*	12a*
			and secondary outcomes	-	-	
			Methods for additional analyses, such as subgroup analyses and adjusted analyses	3	12*	12b*
Results	Participant flow (a diagram is strongly recommended)		For each group, the numbers of participants who were randomly assigned received intended treatment, and were analyzed for the primary outcome	14	13*	13a^†^
			For each group, losses and exclusions after randomization, together with reasons	19		13b^†^
	Recruitment		Dates defining the periods of recruitment and follow-up	-	14	14a*
			Why the trial ended or was stopped		-	14b
	Baseline data		A table showing baseline demographic and clinical characteristics for each group	-	15	15^†^
	Numbers analyzed		For each group, the number of participants (denominator) included in each analysis and whether the analysis was by original assigned groups	16	16*	16^†^
	Outcomes and estimation		For each primary and secondary outcome, results for each group, and the estimated effect size and its precision (such as 95% confidence interval)	15	17*	17a*
	Outcomes and estimation		For binary outcomes, presentation of both absolute and relative effect sizes is recommended	18		17b*
	Ancillary analyses		Results of any other analyses performed, including subgroup analyses and adjusted analyses, distinguishing pre-specified from exploratory	17	18*	18^†^
	Harms		All important harms or unintended effects (for specific guidance see CONSORT for harms)	-	19	19^†^
Discussion	Limitations		Trial limitations, addressing sources of potential bias, imprecision, and, if relevant, multiplicity of analyses	20	20*	20*
	Generalizability		Generalizability (external validity, applicability) of the trial findings		21*	21^†^
	Interpretation		Interpretation consistent with results, balancing benefits and harms, and considering other relevant evidence	21	22*	22*
Other information	Registration		Registration number and name of trial registry	-	-	23
	Protocol		Where the full trial protocol can be accessed, if available	-	-	24
	Funding		Sources of funding and other support (such as supply of drugs), role of funders	-	-	25

*Items were enhanced for specificity of appraisal

^†^Items were simplified and clarified regarding the wording

- indicates the absence of an item

For the 2001 and 2010 CONSORT checklist, the item number in the article is shown. As for the 1996 checklist, there was no item number listed in the article, so item numbers were assigned according to the order shown in the original article

### Characteristics of subject articles/clinical trials

The characteristics of the extracted clinical trials, including the year of publications, number of study arms, blinding, region in which the trial was conducted, number of participant centers, number of enrolled patients, sponsor, and eligible age (individuals aged 18 years or older were classified as adults, whereas those 16 years or older, 13 years or older, and 12 years or older were classified as mixed [adults and children]), were tabulated.

### Evaluation of the quality of reporting in the selected articles

The CONSORT 2010 checklist comprised six sections, 25 topics, and 37 items. If the description of the items was complete, we allocated a score of 1 point to the item, so the full mark was 37 points for an article. If there was no description regarding the item, we scored 0 points. When the reporting of an item was incomplete, it was allocated 0.5 points. Other items using specific scoring methods are defined in the footnotes (points 1–3).

The items scores were totaled for each article. This sum was divided by the number of items which was determined by subtracting the number of irrelevant items excluded from the total number of items. To calculate the reporting rate, this value was expressed as a percentage. In the separate examinations conducted for the three periods, the reporting rates of the individual items were calculated by dividing the total score of the relevant item for articles in which the item was reported by the number of articles extracted in that period, and expressed as a percentage.

Point 1: Reporting rate of 37 items in the CONSORT 2010 checklist (Table 1) [3]

- Item 5 in the CONSORT 2010 checklist (#5).“The interventions for each group are given with sufficient detail to allow replication, including how and when they were actually administered.” We allocated a score of: 0.25 points for the descriptions of the daily dosage and interval; 0.25 points for the description of nutrition, such as fasting, pre-prandial, and postprandial administration; 0.5 points for a detailed description of scheduled visits.
- #11a and #11b. “Blinding.” Cases of non-blinded trials were excluded from the evaluation.
- #14b. “Why the trial ended or was stopped.” Cases in which trial progress could be judged as discontinued/completed/currently enrolled were allocated a score of 1 point.
- #20. “Limitations.” Cases in which the word “limitation” was clearly written were allocated a score of 1 point.

The reporting rate of each section (Title and Abstract, Introduction, Methods, Results, Discussion, and Other information) was calculated by dividing the sum of the percentage of reported items in each section by the number of reported items in each section.

Point 2: Reporting rate for protocol deviations (Fig. 3a)

Sweetman and Doig recommended changes to the CONSORT statement that make reporting requirements for protocol deviations more explicit [10]. They analyzed the relationships between reporting of five individual items in protocol deviations (enrollment, randomization, study intervention, patient compliance, and data collection) and the trial characteristics of 80 articles published in four prominent medical journals [10]. The reporting rates for patient compliance and randomization were the highest and lowest among these items, respectively, although protocol deviations had not been reported frequently. They mentioned that protocol deviations might result in patient harm and introduce errors into clinical trial results. Therefore, we also assessed these items in this study. We allocated 1 point in cases where protocol deviations were clearly reported and 0.5 point in cases where the reporting was incomplete. We then compared our findings with those reported earlier [10].

Point 3: Reporting of ethical aspects

The presence or absence of reporting on obtaining informed consent, acquisition of approval by the ethics committee or institutional review board (IRB) and statements of conflict of interest (COI) were extracted. In terms of COI, cases where “financial disclosures” were stated were allocated 1 point.

### Statistical analysis

The reporting rate per period, expressed as the mean and standard deviation (SD), were compared using a one-way analysis of variance. Additionally, changes in reporting rates of individual evaluation items over the three time periods were examined using the Jonckheere-Terpstra trend test [24]. Since we had 45 items and six categories, we carried out 51 trend tests. A significant level of *p* = 0.05 was divided by the number of tests and adjusted to 0.001 on using the Bonferroni correction for multiple comparisons. R version 3.2.4-revised was used for statistical analyses.

## Results

### Article identified

**Fig. 1:**
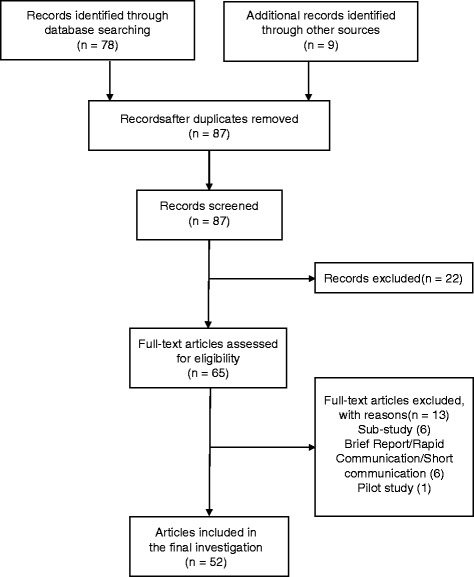
Flowchart of article selection

Seventy-eight articles were identified using the PubMed search. Nine original articles were additionally extracted by manual searches of the reference lists of non-original extracted articles. Abstract review excluded 22 articles from among the 87 non-redundant articles. Of the remaining 65 articles, 13 were excluded after full text review and the remaining 52 articles met the eligibility requirements (Fig. 1) PRISMA checklist is provided in Additional file 1. No articles published before 1996 were found using our search criteria. Table 2 shows the journals in which the selected articles were published.

**Table 2:** Journal impact factors of the current study, as of April 2016

Journal	Current impact factor (2014/2015)	Number of articles (*n* = 52)
*The New England Journal of Medicine*	55.87	7
*The Lancet*	45.22	10
*JAMA: the Journal of the American Medical Association*	35.29	4
*The Lancet Infectious Diseases*	22.43	7
*Annals of Internal Medicine*	17.81	1
*Clinical Infectious Diseases: CID*	8.89	3
*The Journal of Infectious Diseases*	6.00	1
*AIDS*	5.55	9
*Journal of Acquired Immune Deficiency Syndromes: JAIDS*	4.56	4
*PLoS ONE*	3.23	1
*International Medical Press Antiviral Therapy*	3.02	2
*AIDS Research and Human Retroviruses*	2.33	2
*The Lancet HIV* ^a^	0.00	1

^a^Impact factor had not been calculated when the current study was conducted

### Characteristics of selected articles/clinical trials

Most articles identified in our search were published during Period 2 (52%, 27/52), followed by Period 3 (38%, 20/52), and Period 1 (10%, 5/52) Raw data is provided in Additional file 2. Table 3 shows the characteristics of the clinical trials stratified by period. Trials with two arms accounted for approximately 90% of trials, whereas double-blind trials accounted for 40%. Trials conducted in two or more regions accounted for 85% (44/52) of those included. The regions in which the trials were conducted were divided as follows: North America (39/44); Europe (25/44); Oceania (19/44); South America (16/44); Asia (11/44); and Africa (6/44). The median number of study sites was 79.5 (range = 19–234), whereas the median number of enrolled patients was 656.5 (range = 92–2463). The age of 18 years or older as an eligibility criterion was used in approximately 70% (36/52) of trials, whereas trials that allow enrolling younger patients accounted for 20% of the trials. In 10% of the articles, the age of participants was not reported. No trial involved children aged 11 years or younger as participants. Corporations acted as sponsors in 85% (44/52) of trials on average and this percentage increased over the successive time periods (Period 1, Period 2, Period 3 = 60%, 81%, and 95%, respectively). These results show that most of the ART RCTs reported in the selected articles were multicenter international collaborative studies; however, the RCTs conducted in Africa were infrequent.

**Table 3:** Characteristics of included trials

Trial characteristics	Total		Period 1 (1996–2001)		Period 2 (2002–2010)		Period 3 (2011–2016)	
Year of publication	52	-	5	(10%)	27	(52%)	20	(38%)
Number of study arms								
Two	46	(88%)	3	(60%)	24	(89%)	19	(95%)
Three	4	(8%)	1	(20%)	2	(7%)	1	(5%)
Four	2	(4%)	1	(20%)	1	(4%)	0	(0%)
Blinding								
Open-label	29	(56%)	1	(20%)	17	(63%)	11	(55%)
Double-blind	23	(44%)	4	(80%)	10	(37%)	9	(45%)
Region where the trial was conducted								
Europe	3	(6%)	1	(20%)	1	(4%)	1	(5%)
North America	3	(6%)	2	(40%)	0	(0%)	1	(5%)
More than two regions^a^	44	(85%)	2	(40%)	24	(89%)	18	(90%)
Africa	6		0		4		2	
Europe	35		1		18		16	
Asia	11		0		5		6	
North America	39		2		19		18	
South America	16		0		11		5	
Oceania	19		1		7		11	
Not reported	2	(4%)	0	(0%)	2	(7%)	0	(0%)
Number of sites								
Median	79.5		43		78		84.5	
Min	19		28		48		19	
Max	234		81		171		234	
Not reported	8		0		6		2	
Number of patients								
Median	656.5		562		604		687	
Min	92		297		186		92	
Max	2463		2463		1506		1814	
Inclusion criteria of age								
Adult	36	(69%)	1	(20%)	17	(63%)	18	(90%)
Mixed (adults and children)	10	(19%)	2	(40%)	8	(30%)	0	(0%)
Not reported	6	(12%)	2	(40%)	2	(7%)	2	(10%)
Funding								
Academic	5	(10%)	2	(40%)	2	(7%)	1	(5%)
Industry	44	(85%)	3	(60%)	22	(81%)	19	(95%)
Both	1	(2%)	0	(0%)	1	(4%)	0	(0%)
Not reported	2	(4%)	0	(0%)	2	(7%)	0	(0%)

^a^Multiple choice

### Reporting rates based on the CONSORT 2010 checklist for the three periods

**Fig. 2:**
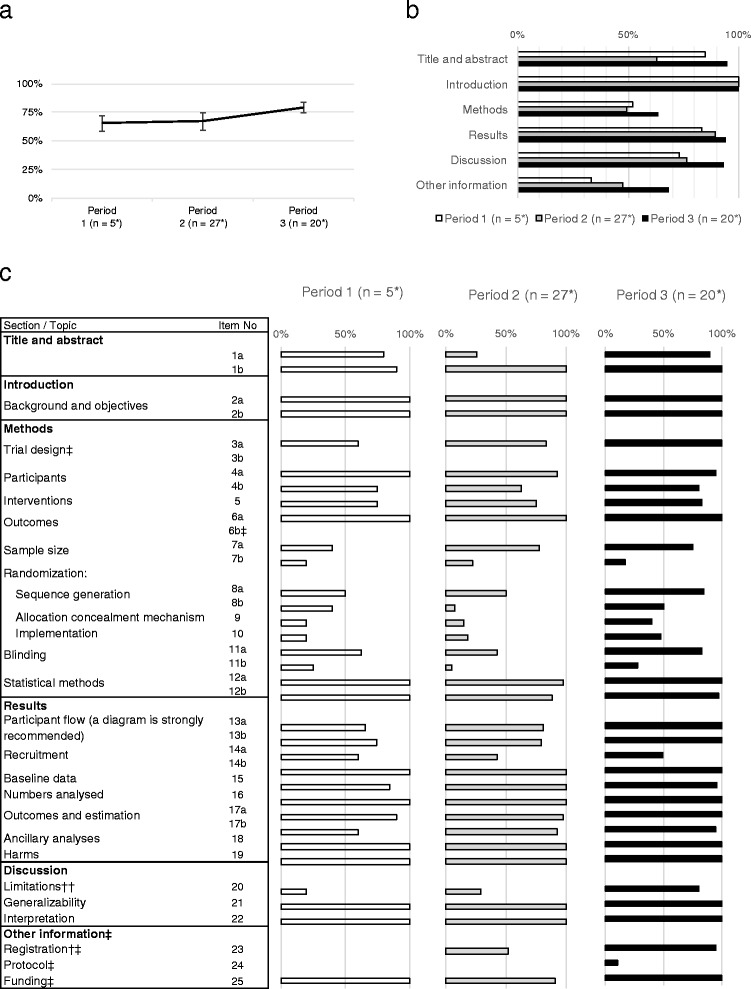
Reporting rate of CONSORT 2010 checklist items among the three periods. **a** Reporting rate for each period. *Bars* show SD. **b** Reporting rate for each section. **c** Reporting rate for each item. * For items 11a and 11b, the number of blinded trials during Periods 1, 2, and 3 were 4, 10, and 9, respectively, and the manuscripts of these trials were examined. † *p* < 0.001, †† *p* = 0.001, ‡ new section and item in the 2010 revision

Figure 2 shows the changes in the reporting rates of individual CONSORT items from Period 1 through Period 3. The overall reporting rate was 71%, whereas the mean reporting rates increased in the three successive periods (65% [SD 7%] in Period 1, 67% [SD 8%] in Period 2, 79% [SD 5%] in Period 3; *p* < 0.0001; Fig. 2a). When considering the reporting rates for the six sections individually, three sections (“Other information” [*p* = 0.109], “Discussion” [*p* = 0.131], “Results” [*p* = 0.430]) tended to increase, but this as not statistically significant (Fig. 2b). The reporting rates of two sections, i.e. “Introduction” (Period 1, Period 2, Period 3 = 100%, 100%, and 100%, respectively) and “Results” (Period 1, Period 2, Period 3 = 83%, 89%, and 94%, respectively) were > 80% in all periods, whereas those of “Methods” (Period 1, Period 2, Period 3 = 52%, 49%, and 63%, respectively) and “Other information” (Period 1, Period 2, Period 3 = 33%, 48%, and 68%, respectively) were < 70% (Fig. 2b). Thus, the reporting rates of many items tended to increase over the revision periods, but the rate was low regardless of the period, as observed for the “Methods” and “Other information” sections.

Among the 37 items, the seven items described below were reported at low rates (<50%). Among the 17 items belonging to the “Methods” section, which had the lowest reporting rate of all sections (Fig. 2c), there were six items: “important changes to methods after trial commencement (such as eligibility criteria), with reasons” (#3b, Period 1, Period 2, Period 3 = 0%, 0%, and 0%, respectively); “any changes to trial outcomes after the trial commenced, with reasons” (#6b, Period 1, Period 2, Period 3 = 0%, 0%, and 0%, respectively); “explanation of any interim analyses and stopping guidelines” (#7b, Period 1, Period 2, Period 3 = 20%, 22%, and 18%, respectively); “mechanism used to implement the random allocation sequence (such as sequentially numbered containers), describing any steps taken to conceal the sequence until interventions were assigned” (#9, Period 1, Period 2, Period 3 = 20%, 15%, and 40%, respectively); “who generated the random allocation sequence, who enrolled participants, and who assigned participants to interventions” (#10, Period 1, Period 2, Period 3 = 20%, 19%, 45%, respectively); and “description of the similarity of intervention in blinding” (#11b, Period 1, Period 2, Period 3 = 25%, 5%, and 28%, respectively). Additionally, “where the full protocol can be accessed, if available” was newly added in the “Other information” section in Period 3 (#24, Period 1, Period 2, Period 3 = 0%, 0%, and 10.0% [ClinicalTrials.gov identifiers: NCT01102972, NCT01263015], respectively). These results show that the items with reporting rates < 50% were “the presence or absence of a protocol change and the reason for such a change,” “randomization and blinding,” and “where the full trial protocol can be accessed.”

When considering the 37 items individually, statistically significant or marginal improvement was seen in two items (“Registration” [*p* < 0.001] and “Limitations” [*p* = 0.001], Fig. 2c). The following nine items had a 100% reporting rate for all periods (“Introduction”: “Background” [#2a] and “Objectives” [#2b]; “Methods”: “Outcomes” [#6a]; “Results”: “Recruitment” [#14b]/“Numbers analyzed” [#16]/“Ancillary analyses” [#18]/“Harms” [#19]; “Discussion”: “Generalizability” [#21]/“Interpretation” [#22]). Among the items newly added in Period 3, “Funding” (#25) retained high reporting rates throughout the periods (Period 1, Period 2, Period 3 = 100%, 91%, and 100%, respectively). “Registration” (#23) was not reported at all in Period 1, but had moderate (52%) and high (95%) rates of reporting in Periods 2 and 3, respectively. Clinical studies involving any intervention are classified into “intervention studies.” In terms of “Interventions” (#5), the daily dose and frequency of administration were reported in all articles, whereas articles in which the temporal relation of medication administration and meals, such as pre-prandial, interprandial, or postprandial administration, accounted for 15% of the selected articles (Period 1, Period 2, Period 3 = n = 0/5, 2/27, and 6/20, respectively). “Harms” (#19) was newly added as an adverse event in Period 2 and exhibited high rates since Period 1 (Fig. 2c). Items with significantly increased reporting rates were the registration in a trial registry and limitations of the trials, and none of the items had a significantly decreased reporting rate. Additionally, information associated with “Harms,” “Background,” and “Objectives” was reported at a high rate, regardless of the period.

### Reporting of protocol deviations

**Fig. 3:**
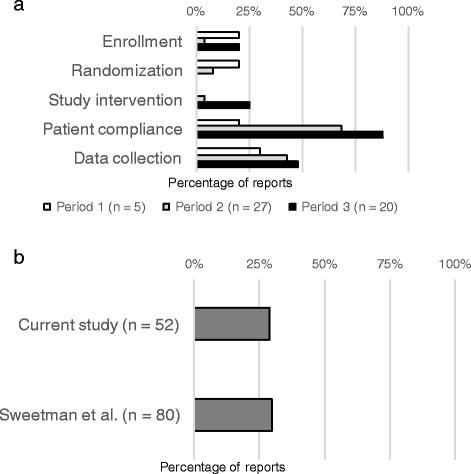
Protocol deviation reporting. **a**: Changes in reporting rates for each period. **b** Difference of total reporting rate between the current study and the previous study

Evaluation of individual items revealed that the rates of reporting on deviations in the study interventions, enrollment, and randomization were < 25%, regardless of the period, and the reporting rate on randomization was the lowest. Additionally, the reporting rates for data collection and patient compliance improved over the successive periods and the rate of patient compliance exceeded 80% in Period 3, revealing a substantial increase (*p* < 0.005, Fig. 3a). The mean 2014/2015 impact factors (IFs) of the articles assessed in this study and the study by Sweetman and Doig were 24.98 and 38.44, respectively. The overall reporting rate of the five items related to protocol deviation was 28.7% in our study and 29.8% in their study; these rates were similar and was < 30% in both studies (Fig. 3b).

### Reporting rates of ethics

**Fig. 4:**
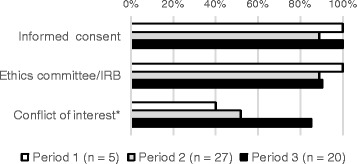
Reporting rates of ethics and conflict of interest. *Including financial disclosure

The rate of reporting on obtaining informed consent (Period 1, Period 2, Period 3 = 100%, 89%, and 100%, respectively) and that on approval by an ethics committee or IRB (Period 1, Period 2, Period 3 = 100%, 89%, and 90%, respectively) remained > 88% throughout the periods (Fig. 4). The rate of reporting on COIs increased over time; it was > 50% in Period 2 and was 84.2% in Period 3 (Fig. 4). In Period 1, financial disclosures rather than COIs were reported in 20% of the articles. Thus, reporting rates for obtaining informed consent and ethical approval were high throughout the study periods, even though the CONSORT checklist did not include either item.

## Discussion

In this study, the quality of reports on RCTs of anti-HIV therapy was evaluated comprehensively and systematically, based on the CONSORT 2010 checklist, protocol deviations, and ethical considerations, which has not been previously reported.

The quality of reported articles evaluated using the CONSORT 2010 checklist improved over time (65% in Period 1, 67% in Period 2, 79% in Period 3; *p* < 0.0001), although seven items had low reporting rates of < 50%, regardless of the study period (“Methods”: 6/17 items; “Other information”: 1/3 items). The six items in the “Methods” section were randomization or blinding (#9, #10, #11b), explanation of any interim analyses and stopping guidelines (#7b), and criteria changes after trial commencement (#3b, #6b). The one item in the “Other information” section was where the full trial protocol can be accessed (#24). Some journals require public posting of protocols as a condition of acceptance for publication [25]. Therefore, it is possible that articles published in the future may improve the reporting rate of item #24.

“Registration” (#23) was not reported at all in Period 1, but the reporting rates in Periods 2 and 3 were moderate (52%) and high (95%), respectively. The International Committee of Medical Journal Editors (ICMJE) requires the registration of clinical trials in a public trials registry. This abrupt increase after the second revision may involve the requirement for registration in Clinicaltrials.gov, etc., since 2000. “Registration” (#23) was added in the CONSORT 2010 revision.

Several studies have reported on compliance and outcomes of studies in the field of HIV/AIDS [13–17] and have demonstrated the close relationship between compliance rates and outcomes. In particular, in this study, the rate after 2010 was very high, at approximately 90%. This reflects the importance of compliance in this disease field. Although both the study by Sweetman and Doig and this study showed low reporting rates (approximately 30%) in overall deviations, the rate can likely be improved by means of a data manager, as management of deviation reporting is within the scope of data management.

In terms of ethical aspects, the rates of reporting on obtaining informed consent and approval by an ethics committee or IRB were high throughout the study period, as previously reported [26]. This is likely because the included trials complied with the Declaration of Helsinki [27], adopted in 1964, and the ICH-GCP guidelines [28], completed in 1996. The rates for reporting on COIs were 40%, 52%, and 85% in Periods 1, 2, and 3, respectively, and thus increased over time. Reporting on COIs was mentioned in the uniform requirements for manuscripts submitted to biomedical journals, published in 1988 [29, 30] by the ICMJE. However, COIs were reported in > 50% of the articles in Period 2, after 2001, indicating that the revision of the COI section in 2001 [31] and the influence of 21 CFR Part 54, described in 1999 [32], were likely to have facilitated reporting of COIs.

This study has several limitations. Although PubMed is one of the largest medical publication databases, we did not evaluate reports that were not indexed in PubMed. Also, we searched for keywords in the Title/Abstracts only and did not search the literature by using relevant subject headings. The second limitation regards scoring. It was simple to score 0 or 1 for about half of the items (17/37 items), but some items could not be determined dichotomously (0 or 1), so we chose intermediate score levels (0.25, 0.5, and 0.75) for some items. This step was subjective in nature. Therefore, we provided a comprehensive description of item scoring in the “Methods” section. We defined the method to evaluate items such as “important changes to methods after trial commencement (such as eligibility criteria), with reasons (#3b)” and “any changes to trial outcomes after the trial commenced, with reasons (#6b)”. But these items are difficult to assess unless the clinical protocol was reviewed. The third limitation is that there is no consensus if it is important to report ethical items for HIV/AIDS RCT articles. We found that earlier reports stating that informed consent required additional effort, due to low literacy rates and differences in language in developing countries [22, 23]. However, we did not find trials involving participants exclusively from sub-Saharan Africa, so we could not assess the difference of reporting rates of ethical items between studies in developing and developed countries. Articles on trials targeting multiple regions accounted for 85% of the individuals included in this study. Although it is likely that, in such cases, informed consent forms were prepared in the language of the respective region, we could not confirm whether there were any problems related to language, as the articles did not report on language issues. The final limitation is that we were unable to find out when the author guidelines of each journal requested CONSORT compliance, so we evaluated all articles consistently under the CONSORT 2010 guideline. There was a possibility of including the journals that did not require authors to follow the CONSORT guidelines.

### Implications for future studies

As mentioned in the background, it is very important to check if the patients take medicine as indicated in the HIV/AIDS RCTs. To our knowledge, this is the first study to perform quantitative evaluation of patient compliance reporting, in addition to the CONSORT checklist items. The increasing trend of adherence to the CONSORT 2010 guideline and the improved reporting rate for patient compliance both imply that the quality of HIV/AIDS RCTs is improving. Our results may assist in further improving the quality of HIV/AIDS RCTs. As for randomization, many study reports referred to the mechanism used to implement the random allocation sequence, but did not refer to the steps taken to conceal the sequence until interventions were assigned, which was required by the CONSORT 2010 statement. By reporting such information on items of low reporting rates, it is expected that the reporting quality of HIV/AIDS RCTs will improve. Additionally, our methods could be applied to RCT reporting in other disease areas, in which patient compliance is critical for evaluation of efficacy and safety of a new drug or a therapy.

## Conclusion

In conclusion, the representative RCT articles in the field of HIV/AIDS examined in this study, had reporting rates for items defined by CONSORT of approximately 70%; these rates additionally improved over time. However, the reporting rate was still low for some items related to randomization or blinding and for the description of changes after the trial commenced. Additionally, protocol deviations were not comprehensively reported.

### Additional files

Additional file 1: PRISMA checklist. (DOC 66 kb)

Additional file 2: Included studies and the information extracted from them. (XLSX 29 kb)

## Appendix 1

PubMed search terms

The initial keywords were as follows:

#1 HIV [Title] AND English [Language]

#2 AND (randomised [Title/Abstract] OR randomized [Title/Abstract] OR randomly assigned [Title/Abstract] OR randomly allocated [Title/Abstract])

#3 AND (Clinical Trial, Phase III [ptyp] OR phase 3[Title/Abstract] OR phase 3b [Title/Abstract] OR noninferiority study [Title/Abstract] OR non-inferiority study [Title/Abstract] OR noninferiority clinical trial [Title/Abstract] OR blinded equivalence study [Title/Abstract] OR multicenter 48-week study [Title/Abstract])

#4 AND (HIV RNA [Title/Abstract] OR HIV-1 RNA [Title/Abstract] OR vRNA [Title/Abstract] OR TLOVR [Title/Abstract] OR virological success [Title/Abstract])

#5 NOT (review [ptyp] OR phase IIb [Title] OR phase 2b [Title] OR retrospective analyses [Title/Abstract] OR drug resistance [Title/Abstract] OR resistance [Title] OR pharmacokinetics [Title] OR supplementation [Title] OR prevention [Title] OR hepatitis [Title/Abstract] OR GM-CSF [Title/Abstract])

## Abbreviations

AE: Adverse event
AIDS: Acquired immune deficiency syndrome
COI: Conflict of interest
CONSORT: Consolidated Standards of Reporting
EC: Ethics committee
HIV: Human immunodeficiency virus
IC: Informed consent
IRB: Institutional review board
PV: Protocol violation
RCT: Randomized controlled trial
STROBE: Strengthening the Reporting of Observational Studies in Epidemiology
UNAIDS: Joint United Nations Programme on HIV/AIDS
